# Pharmacogenomics of Human ABC Transporter ABCC11 (MRP8): Potential Risk of Breast Cancer and Chemotherapy Failure

**DOI:** 10.2174/187152010794473975

**Published:** 2010-10

**Authors:** Yu Toyoda, Toshihisa Ishikawa

**Affiliations:** Graduate School of Bioscience and Biotechnology, Tokyo Institute of Technology, Yokohama 226-8501, Japan

**Keywords:** Apocrine gland, tamoxifen, earwax, estrogen receptor, mastopathy, multidrug resistance, nucleoside, single nucleotide polymorphism (SNP).

## Abstract

Some genetic polymorphisms of human ABC transporter genes are reportedly related to the risk of certain diseases and patients’ responses to medication. Human ABCC11 functions as an ATP-dependent efflux pump for amphipathic anions. One non-synonymous SNP 538G>A (Gly180Arg) has been found to greatly affect the function and stability of *de novo* synthesized ABCC11 (Arg180) variant protein. The SNP variant lacking *N*-linked glycosylation is recognized as a misfolded protein in the endoplasmic reticulum (ER) and readily undergoes proteasomal degradation. This ER-associated degradation of ABCC11 protein underlies the molecular mechanism of affecting the function of apocrine glands. On the other hand, the wild type (Gly180) of ABCC11 is associated with wettype earwax, axillary osmidrosis, colostrum secretion from the mammary gland, and the potential susceptibility of breast cancer. Furthermore, the wild type of ABCC11 reportedly has ability to efflux cyclic nucleotides and nucleoside-based anticancer drugs. The SNP (538G>A) of the *ABCC11* gene is suggested to be a clinical biomarker for prediction of chemotherapeutic efficacy. Major obstacle to the successful chemotherapy of human cancer is development of resistance, and nucleoside-based chemotherapy is often characterized by inter-individual variability. This review provides an overview about the discovery and the genetic polymorphisms in human *ABCC11*. Furthermore, we focus on the impact of *ABCC11* 538G>A on the apocrine phenotype, patients’ response to nucleoside-based chemotherapy, and the potential risk of breast cancer.

## 1 INTRODUCTION

Pharmacogenomics dealing with heredity and response to drugs is increasingly important, since it attempts to explain variability of one or another drug response and to search for the genetic basis of such variations or differences [1-5]. In fact, inter-individual variability in drug response and the emergence of adverse drug reactions are critical issues in drug development as well as in clinical pharmacotherapy. Accumulating evidence strongly suggests that genetic polymorphisms in drug-metabolizing enzymes, transporters, receptors, and other drug targets are linked to inter-individual differences in the efficacy and toxicity of many medications [6-9].

During the past two decades, the role of carrier-mediated transport in determining the pharmacokinetics of drugs has become increasingly evident with the discovery of genetic variants that affect expression and/or function of a given drug transporter [6, 10]. Drug transporters, including ATP-binding cassette (ABC) transporters and solute carrier (SLC) transporters, are expressed at numerous epithelial barriers, such as intestinal epithelial cells, hepatocytes, renal tubular cells, the blood-brain barrier, and cancer cells [10].

The ABC transporters are a family of large proteins in membranes and are able to transport a variety of compounds including metabolites and drugs through membranes at the cost of ATP hydrolysis [11]. Physiological functions of ABC transporters include the transport of lipids, bile salts, toxic compounds, and peptides for antigen presentation or other purposes, such as ion channel-regulation. The human genome contains 48 ABC transporter genes; at least 14 of these are reportedly associated with heritable human diseases [12]. The diseases that are included are rare and heavily transmitted in families. In fact, mutations in ABC transporter genes have been reported to be associated with inherited diseases including Tangier disease T1 (*ABCA1*); Stargardt disease, retinitis pigmentosa and age-related macular degeneration (*ABCA4*); progressive familial intrahepatic cholestasis (*ABCB11*); Dubin–Johnson syndrome (*ABCC2*); pseudoxanthoma elasticum (*ABCC6*); cystic fibrosis (*CFTR/ABCC7*); X-linked adrenoleukodystrophy (*ABCD1* and *ABCD2*); some forms of Zellweger syndrome (*ABCD3* and *ABCD2*), and sitosterolaemia (a rare lipid metabolic disorder inherited as an autosomal recessive trait) (*ABCG5* and *ABCG8*) [11]. Furthermore, it has recently been reported that SNPs in *ABCC11* and *ABCG2* genes are related with axillary osmidrosis [13-15] and gout risk [16-18], respectively. Some additional ABC transporter genes are also implicated in, or are candidates for, other metabolic inherited diseases (http://nutrigene.4t.com:80/humanabc.htm). In this context, mutations and genetic polymorphisms in ABC transporter genes are considered important biomarkers for diagnosis of inherited diseases and prediction of the risk of drug-induced adverse reactions or response to chemotherapy. Among such human ABC transporters, in this review article we will address human ABCC11 to discuss the potential impact of its genetic polymorphisms on the physiological function, breast cancer risk, and patients’ response to nucleoside-based chemotherapy.

## 2 DISCOVERY OF HUMAN *ABCC11* GENE

In 2001, three research groups, including us, independently cloned two novel ABC transporters named ABCC11 and ABCC12 from the cDNA library of human adult liver [19-21]. These two genes have been found to be located on human chromosome 16q12.1 in a tail-to-head orientation with a separation distance of about 20 kb (Fig. (**1A**)). The predicted amino acid sequences of both gene products show a high similarity to those of ABCC4 and ABCC5, suggesting that they have the typical structure of “full” ABC transporter (Fig. (**1B**)). However, there is no putative mouse or rat orthologous gene corresponding to human *ABCC11* [22]. This fact indicates that *ABCC11* is not an orthologous gene but rather a paralogous gene generated by gene duplication in the human genome. On the other hand, *ABCC12* and its orthologous genes are found in different species including humans, primates, and rodents [22].

Transcript analyses suggest that human ABCC11 mRNA is ubiquitously expressed in human adult and fetal tissues [19, 20]. In addition, we [19] and Bera *et al.* [21] demonstrated high levels of ABCC11 mRNA in breast cancer. The increased expression of ABCC11 wild type (WT) in breast cancer might be related with low levels of efficacy of chemotherapy, as discussed later in this review.

When transfected exogenously, the ABCC11 WT protein was localized in the apical membrane of Madin-Darby canine kidney cells strain II (MDCK II) cells [23]. The substrate specificity of ABCC11 WT was characterized in more detail by an *in vitro* transport assay with plasma membrane vesicles prepared from pig LLC-PK1 cells transfected with an ABCC11 WT expression vector [24]. Their assay demonstrated that ABCC11 WT is able to transport a variety of lipophilic anions, including cyclic nucleotides, glutathione conjugates such as leukotriene C_4_ (LTC_4_) and S-(2,4-dinitrophenyl)-glutathione (DNP-SG), steroid sulfates such as estrone 3-sulfate (E_1_3S) and dehydroepiandrostenedione 3-sulphate (DHEAS), glucuronides such as estradiol 17-β-D-glucuronide (E_2_17βG), monoanionic bile acids glycocholate and taurocholate, and folic acid and its analog methotrexate (MTX) (Fig. (**2A**)). Kinetic analyses suggest that cGMP and DHEAS are good substrates for ABCC11 (Table **1**) [23, 24].

## 3 REGULATION OF ABCC11 GENE EXPRESSION

In 2004 Bieche *et al.* [25] reported that ABCC11 was up-regulated in estrogen receptor-α -positive breast tumors, as compared with normal breast tissue. Sarah Park *et al.* [26] investigated the mRNA levels of ABC transporter genes in breast cancer patients who underwent sequential weekly paclitaxel/FEC (5-fluorouracil, epirubicin and cyclophosphamide) neoadjuvant chemotherapy. Their analysis showed that the expression of ABCC11 was increased (fold ratio = 2.71) in the patients with the residual disease as compared with the patients with no pathologic evidence of any residual invasive cancer cells in breast.

More recently, Honorat *et al.* [27] has demonstrated that endogenous ABCC11 mRNA levels in breast cell lines are directly correlated with the estrogen receptor α-status. Interestingly, they found that ABCC11 expression was reduced *in vitro* by estradiol treatments. Furthermore, this estradiol-dependent down-regulation of ABCC11 expression was blocked by co-treatment of tamoxifen (Fig. (**3**)), an antagonist of estradiol. These findings suggest ABCC11 expression is directly or indirectly regulated by estrogen receptor α and that the prolonged exposure of breast cancer cells to tamoxifen can lead to up-regulation of ABCC11.

Hauswald *et al.* [28], on the other hand, have shown that some of histone deacetylase inhibitors induced the expression of several ABC transporters, including *ABCC11* gene, to render acute myeloid leukemia cells a broad-spectrum of drug resistance. Molecular mechanisms underlying the induction remain to be elucidated. Since histone deacetylase inhibitors can be utilized in combination with conventional anti-cancer drugs in clinical trials, such induction of ABCC11 WT may affect the efficacy of nucleoside-based chemotherapy.

## 4 ER-ASSOCIATED DEGRADATION OF ABCC11

ABCC11 WT is an *N*-linked glycosylated protein, which is localized in intracellular granules and large vacuoles as well as at the luminal membrane of secretory cells in the cerumen apocrine gland [14]. *N*-linked glycosylation occurs at both Asn838 and Asn844 in the extracellular loop between transmembrane domains 7 (TM7) and 8 (TM8) of the ABCC11 WT protein. In contrast, the SNP variant: ABCC11 Arg180, which is the determinant of human earwax type (as we see later in this review), lacks *N*-linked glycosylation and readily undergoes proteasomal degradation, most probably via ubiquitination.

The ER and *Golgi* apparatus are the sites of synthesis and maturation of proteins destined for the plasma membrane, for the secretory and endocytic organelles, and for secretion [29, 30]. Efficient quality control systems have evolved to prevent incompletely folded proteins from moving along the secretory pathway. Accumulation of misfolded proteins in the ER would detrimentally affect cellular functions. Therefore, misfolded proteins may be removed from the ER by retrotranslocation to the cytosol compartment where they are degraded by the ubiquitin-proteasome system. This process is known as endoplasmic reticulum-associated degradation (ERAD) [31-34]. It is likely that the SNP variant (Arg180) is recognized as misfolded proteins in the ER and readily undergoes proteasomal degradation. We consider that an electrostatic charge (either positive or negative) at amino acid 180 in transmembrane domain 1 (TM1) interferes with correct folding of *de novo* synthesized ABCC11 protein in the ER [14]. This ERAD processing of the SNP variant (Arg180) of ABCC11 may greatly influence the activity of ceruminous apocrine glands and determine the type of human earwax. Similar ERAD processing is considered to take place for the SNP variant (Arg180) of ABCC11 in axillary and mammary apocrine glands. In Fig. (**4**), we schematically illustrate the impact of the SNP on the cellular localization and function of ABCC11 in secretory cells of the apocrine gland. Asn838 and Asn844 are glycosylation target sites in human ABCC11. The *N*-linked glycans are thought to be subjected to extensive modification as glycoproteins mature and move through the ER via the *Golgi* apparatus to their final destination, for example intracellular granules and large vacuoles of secretory cells in the apocrine gland.

## 5 POLYMORPHISMS/GENETICS

Hitherto more than 10 non-synonymous single nucleotide polymorphisms (SNPs) have been reported in the human *ABCC11* gene (Fig. (**1B**)). Among those SNPs, we have recently found that one SNP (rs17822931; 538G>A, Gly180Arg) determines the human earwax type [35]. Earwax (cerumen) is a secretory product of the ceruminous apocrine glands, which can be classified into two phenotypes in humans, wet (sticky) and dry. The dry type is mostly common in the Asian population, especially in Korean, Japanese, and Chinese, whereas the wet type is a dominant phenotype for many Africans and Caucasians. The AA genotype gives the dry phenotype, whereas both GA and GG genotypes give the wet phenotype. This is consistent with observations that earwax type is a Mendelian trait and that the wet phenotype is dominant to the dry one. 

Interestingly, this SNP exhibits wide ethnic differences in the allele frequency [14]. In Mongoloid populations in Asia, the frequency of the A allele is predominantly high, whereas its allele frequency is low among Caucasians and Africans [14, 35] (Fig. (**5**)). The frequency of the A allele exhibits a north-south and east-west downward geographical gradient with the highest peak in northeastern Asia. It is suggested that the A allele arose in northeast Asia and thereafter spread throughout the world [35], apparently reflecting the inter-continental migration of *Homo sapiens* [14]. A similar west-east downward geographical gradient was observed in the frequency of the 2677G (Ala893) allele of the *ABCB1* (*P-glycoprotein*/*MDR1*) gene as well [36].

## 6 PHYSIOLOGICAL FUNCTION OF ABCC11

Why does one SNP (538G>A) in the human *ABCC11* gene affect the function of apocrine glands? For this question, we have recently provided evidence that proteasomal degradation of the SNP variant (Arg180) of ABCC11 is the underlying molecular mechanism [14]. Immunohistochemical studies with cerumen gland-containing tissue specimens revealed that the ABCC11 WT protein with Gly180 was expressed in the cerumen gland [14]. Interestingly, ABCC11 was predominantly localized in intracellular granules and large vacuoles in the secretory cells of wet-type ceruminous glands. In contrast, such granular and vacuolar localization of ABCC11 was not detected in the dry-type ceruminous glands.

Furthermore, morphological differences were previously reported between the secretory cells of wet and dry types of human ceruminous glands [37]. In the wet-type glands, the *Golgi* apparatus was reportedly well developed, whereas it was generally small in the corresponding cells of the dry type. In addition, intracellular granules were abundantly observed in the wet-type gland in close relationship to their well-developed *Golgi* apparatus, whereas intracellular granules were rare in the dry-type gland.

The cerumen gland is one of the apocrine glands. Apocrine glands can be found not only in the external auditory canal but also in the axillary region and breast; those physical characteristics also are concerned with apocrine glands. In fact, there is a positive association among the wet earwax type, axillary osmidrosis [38], and colostrum secretion from the breast [39]. Therefore, we suppose that ABCC11 WT would regulate the activity and/or development of apocrine glands in human.

## 7 APOCRINE PHENOTYPE

Apocrine secretion occurs when the secretory process is accomplished with a partial loss of cell cytoplasm. The secretory materials may be contained within the secretory vesicles or dissolved in the cytoplasm, and during secretion they are released as cytoplasmic fragments into the glandular lumen or interstitial space [40]. Hitherto apocrine secretory mechanisms have not been well characterized [40]. Although the biochemical and physiological pathways that regulate the apocrine secretory process are not clearly known, our recent findings [13, 14, 35] that the SNP (538G>A, Gly180Arg) in the *ABCC11* gene determines the type of earwax and axillary osmidrosis have shed light on the novel function of this ABC transporter in apocrine glands.

In Japan, axillary osmidrosis is recognized as a disease that is covered by the national health insurance system. Axillary osmidrosis is often perceived, especially by young women, as a distressing and troublesome problem. Certain people display an excessive fear, aversion or psychological hypersensitivity to smells or odors. They tend to opt for aggressive surgical treatments and are sometimes categorized as having osmophobia. 

Sweat produced by axillary apocrine glands is odorless. Secretions from the apocrine glands, however, can be converted to odoriferous compounds by bacteria (Corynebacteria), which results in the formation of the unique “human axillary odor” [41]. In axillary osmidrosis patients (G/G homozygote or G/A heterozygote), significantly numerous and larger-sized axillary apocrine glands were observed as compared with the subjects carrying the A/A homozygote. Indeed, the 538G allele of the *ABCC11* gene is associated with axillary osmidrosis [13-15] and ABCC11 WT (Gly180) is responsible for the secretion of pre-odoriferous compounds from the axillary apocrine gland. In primates, the axillary odors may play a role in olfactory communication, although no documented behavioral or endocrine changes by volatiles produced in the axillae have been reported to occur in humans. Previous studies have shown that androgen steroids were present in the axillary area. Androsterone sulfate (AS) and dehydroepiandrostenedione sulphate (DHEAS) were detected in the extract of axillary hairs, in addition to high levels of cholesterol [42]. It was also demonstrated, following injection of radioactive pregnenolone or progesterone, that steroid secretion was concentrated in the axillary area [43]. The axillary sweat collected in these studies from the skin surface, however, represents a mixture of materials from apocrine, eccrine, and sebaceous glands, in addition to desquamating epidermal cells. In this respect, Labows *et al.* [44] demonstrated that at least two androgen steroids, AS and DHEAS, in addition to cholesterol, did exist in pure apocrine secretions. It is strongly suggested that one of the physiological functions of ABCC11 WT is active transport of steroid metabolites, such as AS and DHEAS, into the lumen of apocrine glands.

## 8 ABCC11 WILD TYPE ALLELE AND BREAST CANCER RISK

In 1971, Nicholas L. Petrakis first reported that international mortality and frequency rates for breast cancer seemed to be associated with the frequency of the allele for wet-type earwax [45]. Caucasians and African-Americans in the USA as well as Germans exhibited approximately four-fold higher rates of breast cancer mortality as compared with Japanese and Taiwanese women [45]. Nevertheless, the phenotypic association of the wet type of earwax with breast cancer has remained to be controversial [45, 46].

At the present time, it is not well understood whether ABCC11 WT really contributes to breast cancer risk. Therefore, we have most recently carried out the genotyping of the SNP 538G>A (Gly180Arg) with a total of 543 Japanese women to examine the association between the frequency rate of breast cancer and the allele frequency of the G allele (WT). Using blood samples from patients with invasive breast cancer (n = 270) and control volunteers (n = 273), we have genotyped the SNP 538G>A in the *ABCC11* gene. The frequency of the G allele in the breast cancer patients was higher than that in the control volunteers. The odds ratio for the genotypes (G/G + G/A) in developing breast cancer was estimated as 1.63 (*p*-value = 0.026), suggesting that the G allele in the *ABCC11* gene is moderately associated with the risk of breast cancer [47]. Genetically-determined variations in the apocrine gland might influence susceptibility to breast cancer. We hypothesize that the function of ABCC11* per se* or metabolites transported by ABCC11 may stimulate the proliferation of apocrine gland cells to enhance the risk of mastopathy (Fig. (**6**)). This hypothesis is supported by evidence that apocrine glands are large in individuals carrying WT allele of A*BCC11* gene. As far as the cell cycle machinery is operating normally, proliferation of apocrine gland cells should stop to certain extent. However, when somatic mutation has occurred in *BRCA1*, *BRCA2*, *p53*, or *p21* genes, it can start deleterious and unregulated proliferation of those cells Fig. (**6**).

## 9 RELEVANCE TO DRUG RESISTANCE IN CANCER CHEMOTHERAPY

It has recently been reported that ABCC11 is potentially involved in drug resistance of breast cancer. ABCC11 mRNA is highly expressed in breast tumors [19, 21, 25], in particular, in invasive ductal adenocarcinomas (https://www.oncomine.org/ resource/logn.html). Its expression is reportedly regulated by estrogen receptor-β [27] and induced by 5-fluorouracil (5-FU) [48]. In addition, it has been reported that ABCC11 is directly involved in 5-FU resistance by the efflux transport of the active metabolite 5-fluoro-2’-deoxyuridine 5’-monophosphate (FdUMP) [48-50]. It is of great interest to investigate whether the expression of ABCC11 WT (538G) is related to drug resistance of breast cancer and high rates of mortality. Further clinical studies, including protein expression studies in tumors, will be needed to clarify the potential contribution of ABCC11 to breast cancer risk and prognosis, including drug resistance and chemosensitivity.

Because of these structural similarities, it could be anticipated that substrate specificities of ABCC11 would be related to ABCC4 and ABCC5. This indeed has been the case. Ectopic expression of ABCC11 in mammalian cells enhances the cellular efflux of cyclic nucleotides and confers resistance to certain anticancer and antiviral nucleotide analogues [50]. In fact, it has been reported that ABCC11 WT has an ability to efflux cyclic nucleotides (*e.g.*, cGMP and cAMP) and confers resistance to several antiviral and anticancer nucleotide analogues, such as 5’-FdUMP and 9’-(2’-phosohonylmethoxynyl)adenine (PMEA) [48-50].

Therapy with nucleoside-derived drugs is characterized by inter-individual variability [51, 52]. Genetic variants that affect protein products involved in all steps leading to drug action maybe major contributors to this heterogeneity of response to nucleoside-based treatments. In particular, variants of drug metabolizing enzymes and transporters might determine the amount of drug needed for an efficient therapeutic response [6].

Successful treatment of cancer remains a therapeutic challenge, with a high percentage of patients suffering from resistance or relapsed disease. One of such examples resides in antileukemic treatment with nucleoside analogues, such as cytarabine (Ara-C) (Fig. (**2B**)). Guo *et al.* have recently presented evidence that expression of ABCC11 WT is an important factor in acute myeloid leukemia patient survival and that the cause of treatment failure in those patients with high expression of ABCC11 WT is very likely an increased extrusion of Ara-C from blast cells mediated by the transporter [53].

Uemura *et al.* have recently found that the gene and protein expression of ABCC11 was higher in pemetrexed (MTA)-resistant cells than in the parental cells [54]. The MTA resistant cells showed cross-resistance to methotrexate (MTX) (Fig. (**2B**)), which is a substrate for ABCC11, and intracellular MTX accumulation in MTA-resistant cells was lower than in the parental cells. They then tested the effect of decreasing the expression of ABCC11 by siRNA and found that decreased expression of ABCC11 enhanced MTA cytotoxicity and increased intracellular MTX accumulation in MTA-resistant cells. These findings suggest that ABCC11 confers resistance to MTA by enhancing efflux of the intracellular anti-cancer drug.

They further analyzed the relationship between *ABCC11* gene expression and MTA sensitivity of 13 adenocarcinoma cell lines. In contrast to their expectation, there was no correlation. Instead, 13 lung adenocarcinoma cell lines could be classified into three groups based on the genotype of the ABCC11 SNP (538G>A): G/G, G/A, and A/A. The A/A group showed a significant reduction in the IC_50_ value of MTA compared with the combined G/G and G/A groups, indicating that the SNP (538G>A) in the *ABCC11* gene is an important determinant of MTA sensitivity. These results suggest that the SNP (538G>A) of the *ABCC11* gene may be one of the biomarkers for MTA treatment in adenocarcinomas.

## CONCLUSIONS

In this review article, we have addressed the potential impact of *ABCC11* 538G>A on the apocrine phenotype, patients’ response to nucleoside-based chemotherapy, and the potential risk of breast cancer. However, it should be carefully evaluated by clinical studies whether the SNP (538G>A) of the *ABCC11* gene is a clinically important biomarker for prediction of chemotherapeutic efficacy. In addition, we need to further elucidate whether there are any other diseases that involve apocrine secretion and to explore the clinical significance of ABCC11.

Pharmacogenomic studies are rapidly elucidating the inherited nature of differences in pharmacokinetic and pharmacodynamic effects, thereby enhancing drug discovery and providing a stronger scientific basis for optimizing drug therapy on the basis of each patient’s genetic constitution. For the next step, therefore, development of new technology (e.g., rapid, accurate, and cost-effective diagnosis methods) is needed to facilitate clinical research of pharamacogenomics and to support genotyping-based personalized medicine.

## Figures and Tables

**Fig. (1):**
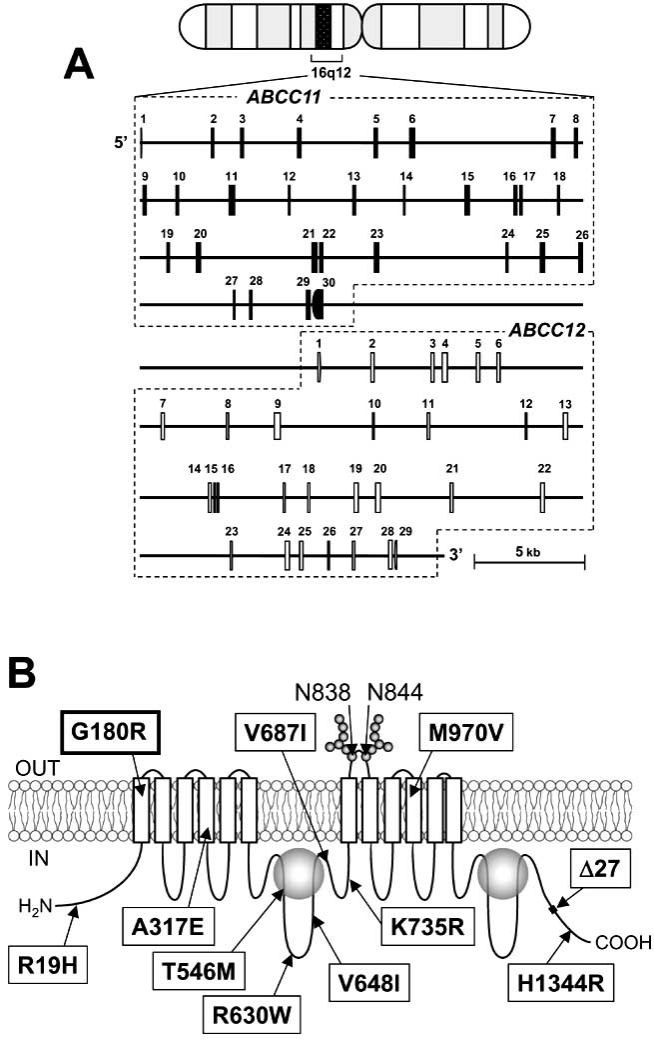
(**A**) The genomic structures of *ABCC11* and *ABCC12* genes on human chromosome 16q12.1. The cytogenetic location of the *ABCC11* gene as well as the structures of exons and introns were analysed by BLAST searches on the human genome. The *ABCC11* gene is encoded by a -68 kb gene consisting of 30 exons. A non-synonymous SNP: 538G>A (Gly180Arg), an earwax determinant, is in the exon 4 of *ABCC11* gene. (**B**) Schematic illustration of ABCC11 structure and hitherto known non-synonymous SNPs. ABCC11 has a total of 12 transmembrane (TM) regions and two intracellular ATP-binding cassettes. Asn838 and Asn844 residing in an extracellular loop between transmembrane helices TM7 and TM8 are *N*-linked glycosylation sites in the ABCC11 WT protein. Locations of hitherto reported nonsynonymous SNPs and Δ27 (rare deletion mutation) are indicated in the putative structure of ABCC11. G180R and Δ27 are related with the formation of dry-type earwax.

**Fig. (2):**
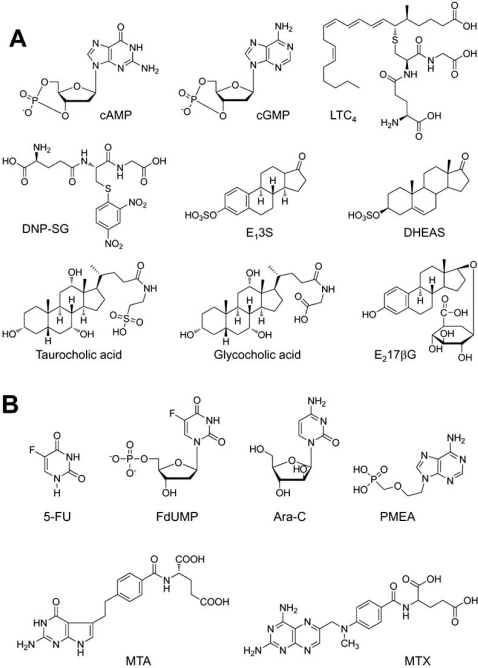
(**A**). Chemical structures of classical substrates of ABCC11. cAMP (cyclic adenosine monophosphate), cGMP (cyclic guanosine monophosphate), LTC_4_ (leukotriene C_4_), DNP-SG (S-(2,4-dinitrophenyl)-glutathione), E_1_3S (estrone 3-sulfate), DHEAS (dehydroepiandrosterone 3-sulfate), and E_2_17βG (estradiol 17-β-D-glucuronide). (**B**) Anticancer drugs that are transported by ABCC11. MTA (pemetrexed), MTX, (methotrexate), Ara-C (Cytosine arabinoside), PMEA (9’-(2’-phosphonyl-methoxyethyl)adenine) are substrate for ABCC11. 5-fluoro-2’-deoxyuridine 5’-monophosphate (FdUMP) is an active metabolite of 5-FU (5-fluorouracil) and transported by ABCC11.

**Fig. (3):**
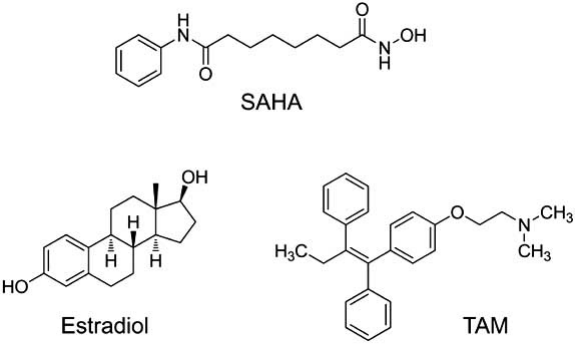
Anticancer drugs that regulate the expression of ABCC11. SAHA (suberoylanilide hydroxamic acid), one of the histone deacetylase inhibitors, induces the expression of ABCC11 mRNA. Estradiol reduces the expression of ABCC11 transcript in estrogen receptor-α-positive breast cancer cells, whereas TAM (tamoxifen), an estrogen receptor-α antagonist, abrogates the estrogen-mediated down-regulation effect.

**Fig. (4):**
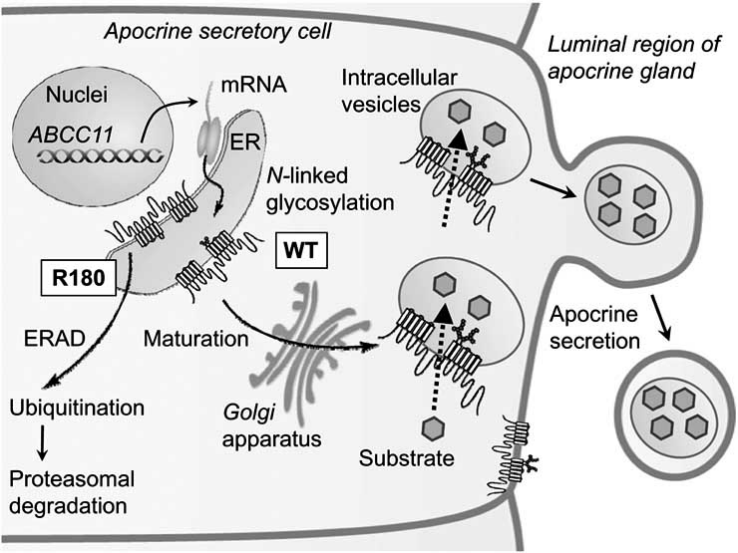
Schematic illustration of intracellular sorting of ABCC11 WT and proteasomal degradation of the R180 (Arg180) variant in secretory cells of the ceruminous apocrine gland. *De novo* synthesized ABCC11 WT is *N*-linked glycosylated at Asn838 and Asn844 in the ER, further processed in the *Golgi* apparatus, and destined for membrane of intracellular granules and vacuoles. Ceruminous components are thought to be transported by ABCC11 WT and sequestrated in intracellular granules and vacuoles. SNP variant R180 lacking *N*-linked glycosylation is recognized as misfolded proteins in the ER and readily undergo ubiquitination and proteasomal degradation (ERAD pathway). ER, endoplasmic reticulum; ERAD, ER-associated degradation.

**Fig. (5):**
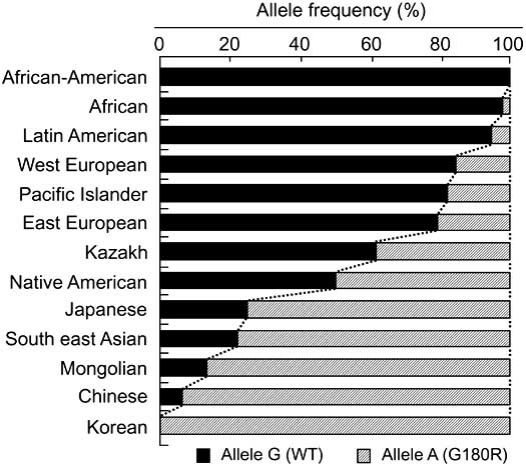
The allele frequencies of the wild type (WT, Gly180) and the 538G>A (Arg180) variant of human ABCC11 among different ethnic populations. Data are from Yoshiura *et al*. [25].

**Fig. (6):**
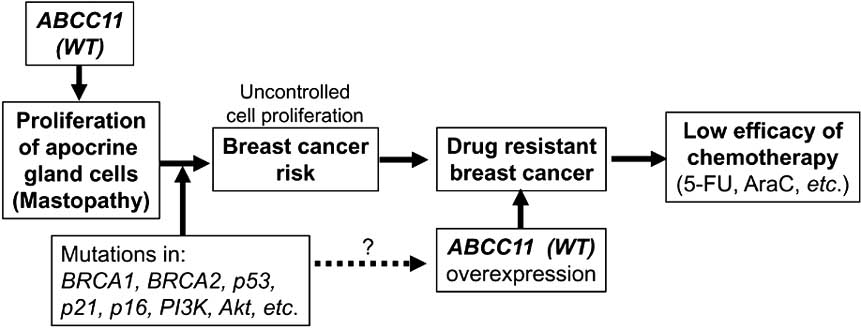
The potential impact of *ABCC11* WT (538G) on the apocrine phenotype, patients’ response to nucleoside-based chemotherapy, and the risk of mastopathy and breast cancer.

**Table 1:** Kinetic Parameters for ABCC11-Mediated Transport

Substrates	Km [µM]	Vmax [pmol/mg/min]	Reference
cGMP	7.8	-	Yoshiura *et al.* 2005 [35]
DHEAS	13.0	34.9	Chen *et al.* 2005 [24]
	21.0	370.0	Bortfeld *et al.* 2006 [23]
E_2_17βG	62.9	62.0	Chen *et al.* 2005 [24]
E_1_S3	150.0	-	Bortfeld *et al.* 2006 [23]
MTX	957.0	317.0	Chen *et al.* 2005 [24]

## References

[1] Weiss S T, McLeod H L, Flockhart D A, Dolan M E, Benowitz N L, Johnson J A, Ratain M J, Giacomini K M (2008). Creating and evaluating genetic tests predictive of drug response. Nat. Rev. Drug Discov.

[2] Sadee W (2008). Drug therapy and personalized health care: pharmacogenomics in perspective. Pharm. Res.

[3] Wilke R A, Lin D W, Roden D M, Watkins P B, Flockhart D, Zineh I, Giacomini K M, Krauss R M (2007). Identifying genetic risk factors for serious adverse drug reactions: current progress and challenges. Nat. Rev. Drug Discov.

[4] Andersson T, Flockhart D A, Goldstein D B, Huang S M, Kroetz D L, Milos P M, Ratain M J, Thummel K (2005). Drug-metabolizing enzymes: evidence for clinical utility of pharmacogenomic tests. Clin. Pharmacol. Ther.

[5] Kalow W, Meyer U, Tyndale R F (2001). Pharmacogenomics.

[6] Errasti-Murugarren E, Pastor-Anglada M (2010). Drug transporter pharmacogenetics in nucleoside-based therapies. Pharmacogenomics.

[7] Cusatis G, Sparreboom A (2008). Pharmacogenomic importance of ABCG2. Pharmacogenomics.

[8] Ishikawa T, Tsuji A, Inui K, Sai Y, Anzai N, Wada M, Endou H, Sumino Y (2004). The genetic polymorphism of drug transporters: functional analysis approaches. Pharmacogenomics.

[9] Kim R B (2002). Pharmacogenetics of CYP enzymes and drug transporters: remarkable recent advances. Adv. Drug Deliv. Rev.

[10] Giacomini K M, Huang S M, Tweedie D J, Benet L Z, Brouwer K L, Chu X, Dahlin A, Evers R, Fischer V, Hillgren K M, Hoffmaster K A, Ishikawa T, Keppler D, Kim R B, Lee C A, Niemi M, Polli J W, Sugiyama Y, Swaan P W, Ware J A, Wright S H, Yee S W, Zamek-Gliszczynski M J, Zhang L (2010). Membrane transporters in drug development. Nat. Rev. Drug Discov.

[11] Holland I, Cole S P C, Kuchler K, Higgins C F (2002). ABC Protein: From Bacteria to Man.

[12] Borst P, Elferink R O (2002). Mammalian ABC transporters in health and disease. Annu. Rev. Biochem.

[13] Inoue Y, Mori T, Toyoda Y, Sakurai A, Ishikawa T, Mitani Y, Hayashizaki Y, Yoshimura Y, Kurahashi H, Sakai Y (2010). Correlation of axillary osmidrosis to a SNP in the ABCC11 gene determined by the Smart Amplification Process (SmartAmp) method. J. Plast. Reconstr. Aesthet. Surg.

[14] Toyoda Y, Sakurai A, Mitani Y, Nakashima M, Yoshiura K, Nakagawa H, Sakai Y, Ota I, Lezhava A, Hayashizaki Y, Niikawa N, Ishikawa T (2009). Earwax, osmidrosis, and breast cancer: why does one SNP (538G>A) in the human ABC transporter ABCC11 gene determine earwax type?. FASEB J.

[15] Nakano M, Miwa N, Hirano A, Yoshiura K, Niikawa N (2009). A strong association of axillary osmidrosis with the wet earwax type determined by genotyping of the ABCC11 gene. BMC Genet.

[16] Wang B, Miao Z, Liu S, Wang J, Zhou S, Han L, Meng D, Wang Y, Li C, Ma X (2010). Genetic analysis of ABCG2 gene C421A polymorphism with gout disease in Chinese Han male population. Hum. Genet.

[17] Woodward O M, Kottgen A, Coresh J, Boerwinkle E, Guggino W B, Kottgen M (2009). Identification of a urate transporter, ABCG2, with a common functional polymorphism causing gout. Proc. Natl. Acad. Sci. USA.

[18] Matsuo H, Takada T, Ichida K, Nakamura T, Nakayama A, Ikebuchi Y, Ito K, Kusanagi Y, Chiba T, Tadokoro S, Takada Y, Oikawa Y, Inoue H, Suzuki K, Okada R, Nishiyama J, Domoto H, Watanabe S, Fujita M, Morimoto Y, Naito M, Nishio K, Hishida A, Wakai K, Asai Y, Niwa K, Kamakura K, Nonoyama S, Sakurai Y, Hosoya T, Kanai Y, Suzuki H, Hamajima N, Shinomiya N (2009). Common defects of ABCG2, a high-capacity urate exporter, cause gout: a function-based genetic analysis in a Japanese population. Sci. Transl. Med.

[19] Yabuuchi H, Shimizu H, Takayanagi S, Ishikawa T (2001). Multiple splicing variants of two new human ATP-binding cassette transporters, ABCC11 and ABCC12. Biochem. Biophys. Res. Commun.

[20] Tammur J, Prades C, Arnould I, Rzhetsky A, Hutchinson A, Adachi M, Schuetz J D, Swoboda K J, Ptacek L J, Rosier M, Dean M, Allikmets R (2001). Two new genes from the human ATP-binding cassette transporter superfamily, ABCC11 and ABCC12, tandemly duplicated on chromosome 16q12. Gene.

[21] Bera T K, Lee S, Salvatore G, Lee B, Pastan I (2001). MRP8, a new member of ABC transporter superfamily, identified by EST database mining and gene prediction program, is highly expressed in breast cancer. Mol. Med.

[22] Shimizu H, Taniguchi H, Hippo Y, Hayashizaki Y, Aburatani H, Ishikawa T (2003). Characterization of the mouse Abcc12 gene and its transcript encoding an ATP-binding cassette transporter, an orthologue of human ABCC12. Gene.

[23] Bortfeld M, Rius M, Konig J, Herold-Mende C, Nies A T, Keppler D (2006). Human multidrug resistance protein 8 (MRP8/ABCC11), an apical efflux pump for steroid sulfates, is an axonal protein of the CNS and peripheral nervous system. Neuroscience.

[24] Chen Z S, Guo Y, Belinsky M G, Kotova E, Kruh G D (2005). Transport of bile acids, sulfated steroids, estradiol 17-beta-D-glucuronide, and leukotriene C4 by human multidrug resistance protein 8 (ABCC11). Mol. Pharmacol.

[25] Bieche I, Girault I, Urbain E, Tozlu S, Lidereau R (2004). Relationship between intratumoral expression of genes coding for xenobiotic-metabolizing enzymes and benefit from adjuvant tamoxifen in estrogen receptor alpha-positive postmenopausal breast carcinoma. Breast Cancer Res.

[26] Park S, Shimizu C, Shimoyama T, Takeda M, Ando M, Kohno T, Katsumata N, Kang Y K, Nishio K, Fujiwara Y (2006). Gene expression profiling of ATP-binding cassette (ABC) transporters as a predictor of the pathologic response to neoadjuvant chemotherapy in breast cancer patients. Breast Cancer Res. Treat.

[27] Honorat M, Mesnier A, Vendrell J, Guitton J, Bieche I, Lidereau R, Kruh G D, Dumontet C, Cohen P, Payen L (2008). ABCC11 expression is regulated by estrogen in MCF7 cells, correlated with estrogen receptor alpha expression in postmenopausal breast tumors and overexpressed in tamoxifen-resistant breast cancer cells. Endocr. Relat. Cancer.

[28] Hauswald S, Duque-Afonso J, Wagner M M, Schertl F M, Lubbert M, Peschel C, Keller U, Licht T (2009). Histone deacetylase inhibitors induce a very broad, pleiotropic anticancer drug resistance phenotype in acute myeloid leukemia cells by modulation of multiple ABC transporter genes. Clin. Cancer Res.

[29] Helenius A, Aebi M (2004). Roles of N-linked glycans in the endoplasmic reticulum. Annu. Rev. Biochem.

[30] Ellgaard L, Molinari M, Helenius A (1999). Setting the standards: quality control in the secretory pathway. Science.

[31] Kleizen B, Braakman I (2004). Protein folding and quality control in the endoplasmic reticulum. Curr. Opin. Cell Biol.

[32] Hampton R Y (2002). ER-associated degradation in protein quality control and cellular regulation. Curr. Opin. Cell Biol.

[33] Ellgaard L, Helenius A (2001). ER quality control: towards an understanding at the molecular level. Curr. Opin. Cell Biol.

[34] Mori K (2000). Tripartite management of unfolded proteins in the endoplasmic reticulum. Cell.

[35] Yoshiura K, Kinoshita A, Ishida T, Ninokata A, Ishikawa T, Kaname T, Bannai M, Tokunaga K, Sonoda S, Komaki R, Ihara M, Saenko V A, Alipov G K, Sekine I, Komatsu K, Takahashi H, Nakashima M, Sosonkina N, Mapendano C K, Ghadami M, Nomura M, Liang D S, Miwa N, Kim D K, Garidkhuu A, Natsume N, Ohta T, Tomita H, Kaneko A, Kikuchi M, Russomando G, Hirayama K, Ishibashi M, Takahashi A, Saitou N, Murray J C, Saito S, Nakamura Y, Niikawa N (2006). A SNP in the ABCC11 gene is the determinant of human earwax type. Nat. Genet.

[36] Sakurai A, Onishi Y, Hirano H, Seigneuret M, Obanayama K, Kim G, Liew E L, Sakaeda T, Yoshiura K, Niikawa N, Sakurai M, Ishikawa T (2007). Quantitative structure--activity relationship analysis and molecular dynamics simulation to functionally validate nonsynonymous polymorphisms of human ABC transporter ABCB1 (P-glycoprotein/MDR1). Biochemistry.

[37] Shugyo Y, Sudo N, Kanai K, Yamashita T, Kumazawa T, Kanamura S (1988). Morphological differences between secretory cells of wet and dry types of human ceruminous glands. Am. J. Anat.

[38] Yoo W M, Pae N S, Lee S J, Roh T S, Chung S, Tark K C (2006). Endoscopy-assisted ultrasonic surgical aspiration of axillary osmidrosis: a retrospective review of 896 consecutive patients from 1998 to 2004. J. Plast. Reconstr. Aesthet. Surg.

[39] Miura K, Yoshiura K, Miura S, Shimada T, Yamasaki K, Yoshida A, Nakayama D, Shibata Y, Niikawa N, Masuzaki H (2007). A strong association between human earwax-type and apocrine colostrum secretion from the mammary gland. Hum. Genet.

[40] Gesase A P, Satoh Y (2003). Apocrine secretory mechanism: recent findings and unresolved problems. Histol. Histopathol.

[41] Shehadeh N H, Kligman A M (1963). The effect of topical antibacterial agents on the bacterial flora of the axilla. J. Invest. Dermatol.

[42] Julesz M (1968). New advances in the field of androgenic steroidogenesis of the human skin. Acta Med. Acad. Sci. Hung.

[43] Brooksbank B W (1970). Labelling of steroids in axillary sweat after administration of 3H-delta-5-pregnenolone and 14C-progesterone to a healthy man. Experientia.

[44] Labows J N, Preti G, Hoelzle E, Leyden J, Kligman A (1979). Steroid analysis of human apocrine secretion. Steroids.

[45] Petrakis N L (1971). Cerumen genetics and human breast cancer. Science.

[46] Ing R, Petrakis L, Ho H C (1973). Evidence against association between wet cerumen and breast cancer. Lancet.

[47] Ota I, Sakurai A, Toyoda Y, Morita A, Sasaki T, Chishima T, Yamakado M, Kawai Y, Ishidao T, Lezhava A, Yoshiura K, Yogo S, Hayashizaki Y, Ishikawa T, Ishikawa T, Endo I, Ishimada H (2011). Association Between Breast Cancer Risk and the Wild-Type Allele of Human ABC Transporter ABCC11. Anticancer Res.

[48] Oguri T, Bessho Y, Achiwa H, Ozasa H, Maeno K, Maeda H, Sato S, Ueda R (2007). MRP8/ABCC11 directly confers resistance to 5-fluorouracil. Mol. Cancer Ther.

[49] Kruh G D, Guo Y, Hopper-Borge E, Belinsky M G, Chen Z S (2007). ABCC10, ABCC11, and ABCC12. Pflugers. Arch.

[50] Guo Y, Kotova E, Chen Z S, Lee K, Hopper-Borge E, Belinsky M G, Kruh G D (2003). MRP8, ATP-binding cassette C11 (ABCC11), is a cyclic nucleotide efflux pump and a resistance factor for fluoropyrimidines 2',3'-dideoxycytidine and 9'-(2'-phosphonylmethoxyethyl)adenine. J. Biol. Chem.

[51] Abbruzzese J L, Grunewald R, Weeks E A, Gravel D, Adams T, Nowak B, Mineishi S, Tarassoff P, Satterlee W, Raber M N, Plunkett W (1991). A phase I clinical, plasma, and cellular pharmacology study of gemcitabine. J. Clin. Oncol.

[52] Heinemann V, Hertel L W, Grindey G B, Plunkett W (1988). Comparison of the cellular pharmacokinetics and toxicity of 2',2'-difluorodeoxycytidine and 1-beta-D-arabinofuranosylcytosine. Cancer Res.

[53] Guo Y, Kock K, Ritter C A, Chen Z S, Grube M, Jedlitschky G, Illmer T, Ayres M, Beck J F, Siegmund W, Ehninger G, Gandhi V, Kroemer H K, Kruh G D, Schaich M (2009). Expression of ABCC-type nucleotide exporters in blasts of adult acute myeloid leukemia: relation to long-term survival. Clin. Cancer Res.

[54] Uemura T, Oguri T, Ozasa H, Takakuwa O, Miyazaki M, Maeno K, Sato S, Ueda R (2010). ABCC11/MRP8 confers pemetrexed resistance in lung cancer. Cancer Sci.

